# Using the Reactome Database

**DOI:** 10.1002/cpz1.722

**Published:** 2023-04-13

**Authors:** Karen Rothfels, Marija Milacic, Lisa Matthews, Robin Haw, Cristoffer Sevilla, Marc Gillespie, Ralf Stephan, Chuqiao Gong, Eliot Ragueneau, Bruce May, Veronica Shamovsky, Adam Wright, Joel Weiser, Deidre Beavers, Patrick Conley, Krishna Tiwari, Bijay Jassal, Johannes Griss, Andrea Senff‐Ribeiro, Timothy Brunson, Robert Petryszak, Henning Hermjakob, Peter D'Eustachio, Guanming Wu, Lincoln Stein

**Affiliations:** ^1^ Ontario Institute for Cancer Research Toronto Ontario Canada; ^2^ NYU Langone Health New York New York; ^3^ European Molecular Biology Laboratory European Bioinformatics Institute (EMBL‐EBI) Wellcome Genome Campus Hinxton Cambridgeshire UK; ^4^ College of Pharmacy and Health Sciences St. John's University Queens New York; ^5^ Oregon Health and Science University Portland Oregon; ^6^ Department of Dermatology Medical University of Vienna Vienna Austria; ^7^ Universidade Federal do Paraná Curitiba Brazil; ^8^ Department of Molecular Genetics University of Toronto Toronto Ontario Canada

**Keywords:** biological pathway, interaction network, pathway analysis, pathway visualization, Reactome database

## Abstract

Pathway databases provide descriptions of the roles of proteins, nucleic acids, lipids, carbohydrates, and other molecular entities within their biological cellular contexts. Pathway‐centric views of these roles may allow for the discovery of unexpected functional relationships in data such as gene expression profiles and somatic mutation catalogues from tumor cells. For this reason, there is a high demand for high‐quality pathway databases and their associated tools. The Reactome project (a collaboration between the Ontario Institute for Cancer Research, New York University Langone Health, the European Bioinformatics Institute, and Oregon Health & Science University) is one such pathway database. Reactome collects detailed information on biological pathways and processes in humans from the primary literature. Reactome content is manually curated, expert‐authored, and peer‐reviewed and spans the gamut from simple intermediate metabolism to signaling pathways and complex cellular events. This information is supplemented with likely orthologous molecular reactions in mouse, rat, zebrafish, worm, and other model organisms. © 2023 The Authors. Current Protocols published by Wiley Periodicals LLC.

**Basic Protocol 1**: Browsing a Reactome pathway

**Basic Protocol 2**: Exploring Reactome annotations of disease and drugs

**Basic Protocol 3**: Finding the pathways involving a gene or protein

**Alternate Protocol 1**: Finding the pathways involving a gene or protein using UniProtKB (SwissProt), Ensembl, or Entrez gene identifier

**Alternate Protocol 2**: Using advanced search

**Basic Protocol 4**: Using the Reactome pathway analysis tool to identify statistically overrepresented pathways

**Basic Protocol 5**: Using the Reactome pathway analysis tool to overlay expression data onto Reactome pathway diagrams

**Basic Protocol 6**: Comparing inferred model organism and human pathways using the Species Comparison tool

**Basic Protocol 7**: Comparing tissue‐specific expression using the Tissue Distribution tool

## INTRODUCTION

The availability of whole genome sequences from numerous species coupled with an explosion of techniques for querying and analyzing these reference genomes, including at a single cell level, has led to a high demand for sophisticated tools to facilitate visualization and interpretation of the resulting large data sets. Biological pathway databases are uniquely positioned to play a key role in the interpretation of such data sets. Pathway databases capture what is already known about the interplay of genes, proteins, and small molecules using a data model that is accessible to computation, and position experimental outcomes on proteins or other biological molecules in their relevant cellular context. For example, a perturbation experiment that changes the expression pattern of thousands of genes may only affect the expression patterns of a small handful of biochemical pathways. Pathway analysis has the potential to reveal unexpected connections between disparate areas of biology that are not readily apparent by simple inspection. Hence, there is a high degree of interest in the bioinformatics community in creating pathway databases. The Reactome project, covered in this article, is one such database. It is a curated collection of well‐documented human molecular reactions grouped into pathways that span the gamut from simple intermediary metabolism (e.g., sugar catabolism) to complex cellular events such as the mitotic cell cycle. Reactome annotations also document how normal biological pathways are affected during disease, and the effects of drugs on pathway activities. Reactome annotations are manually curated by PhD level scientists and peer‐reviewed by experts in the field prior to being published in the database. A semi‐automated procedure supplements this manually curated information by identifying likely orthologous molecular reactions in mouse, rat, zebrafish, worm, and other model organisms, extending the use of the database to support research in other species.

The protocols in this article illustrate how to use Reactome to learn the steps of a biological pathway and how a suite of data analysis tools can assist with the interpretation of user‐supplied experimental data sets. Basic Protocol 1 describes how to navigate and browse through the Reactome database. Basic Protocol 2 describes how to navigate and browse through the drug and disease annotations of Reactome. Basic Protocol 3 and Alternate Protocol 1 explain how to identify the pathways in which a molecule of interest is involved using either the common name or accession number, respectively. Alternate Protocol 2 describes how to use the Advanced Search Feature. Basic Protocol 4 details how to use Pathway Analysis to perform identifier mapping and overrepresentation analysis. Basic Protocol 5 explains how to overlay pathway diagrams with expression data. Basic Protocol 6 describes the use of the Species Comparison tool to compare model organisms and human pathways. Basic Protocol 7 describes how to compare expression in different tissues using the Tissue Distribution tool.

*NOTE*: This information is based on Reactome in December 2022. Some of the web pages may have changed somewhat since the article was written.

## Basic Protocol 1 BROWSING A REACTOME PATHWAY

This protocol will introduce the basic navigational techniques needed to browse the Reactome Web site.

### Necessary Resources

#### Hardware

Computer capable of supporting a Web browser and an Internet connection

##### Software

Any modern Web browser such as Firefox, Safari, and Chrome will work to display Reactome Web pages

**Figure 1:**
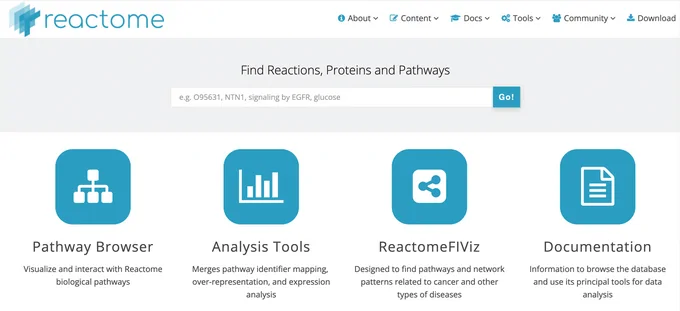
The Reactome home page (https://reactome.org) features a header panel with drop‐down menus to access Web content, a search bar and four large buttons linking to key features of the Web site: Pathway Browser, Analysis Tools, the Reactome FIViz app, and documentation.

**Figure 2:**
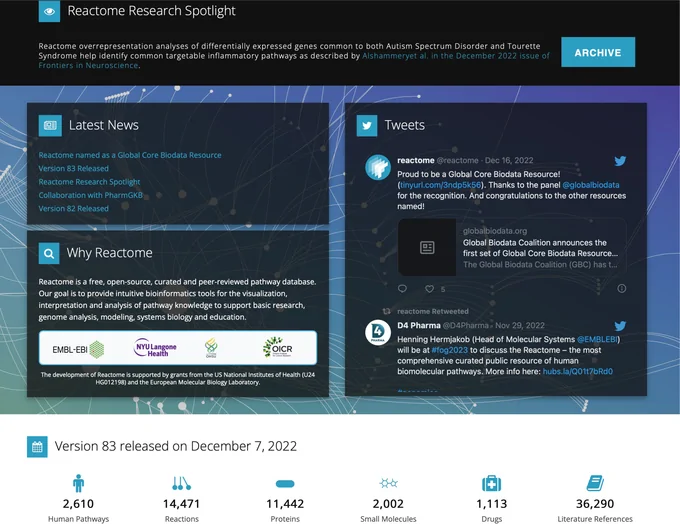
The Reactome home page also contains announcements (news, Twitter, research Spotlight, project information), and statistics from the most recent release.

**Figure 3:**
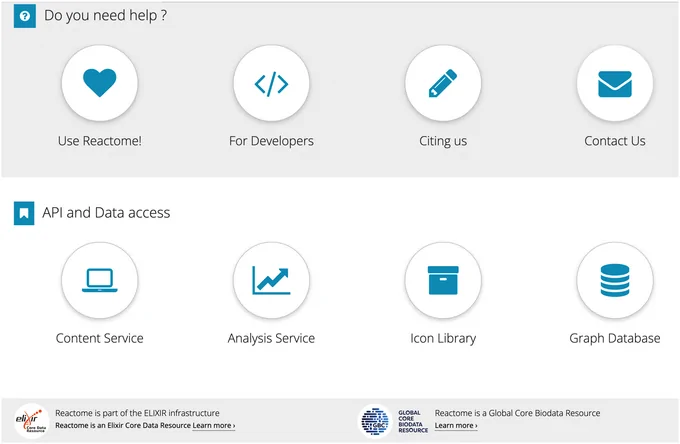
Lower down on the home page is a help panel with buttons linking to guides for users and developers, and buttons for API and data access.

1 Point the browser to the Reactome home page at https://reactome.org.
  The home page (Fig. 1) has several elements.
  At the top left of the home page is the Reactome logo. Clicking on this from any page on the Web site will return the user to the home page.
  The **navigation bar,** at the very top of the page, provides access to the top‐level sections, tools, and resources of the Reactome site. “About” is a description of the project as a whole, including Reactome team and Scientific Advisory Board members, details of our open source licenses, the upcoming editorial calendar and current statistics; “Content” provides links to resources within the database, including the table of contents, database object identifiers, a detailed description of the Reactome data schema as well as information on specific features such as the Reactome Research Spotlight, the COVID‐19 Disease project and the ORCID integration project; “Docs” provides access to user guides and information about the Reactome data model, icon library and computationally inferred events (described more fully in step 3, below), as well as instructions on how to link to or cite Reactome material; “Tools” provides links to key Reactome functions, including the pathway browser, tools for analyzing gene lists and gene expression data, for species comparisons, tissue distribution and for disease overlay. This tab also has links to the Reactome analysis and content services and Reactome FIViz, the Reactome functional interaction network app (Wu et al., 2014). “Community” has information on outreach and events, Reactome publications, partnerships and collaborations as well as access to training guides and tutorials; “Download” provides access to the whole database as a single bulk or individual data set download, pathway downloads in a variety of formats including BioPAX, SMBL, and PDF, as well as physical entity and event mapping files.
  Below the header is a simple search bar that permits flexible keyword, accession number, and database identifier queries on the Reactome database. Below the search bar are four large buttons linking to key features of the Reactome Web site: “Pathway Browser”, which takes the user into the curated pathway hierarchy of human biological pathways (described in step 2 below), “Analysis Tools”, which opens the analysis window allowing users to analyze gene lists and expression patterns and to conduct species comparisons and examine tissue distribution, “ReactomeFIViz”, which takes users to the documentation page for the Reactome functional interaction network app (Wu et al., 2014), and “Documentation”, which links to useful information about the Web site for users and developers.
  Below these buttons is a section of the home page (Fig. 2) that contains the “Reactome Research Spotlight,” (a feature that highlights recent publications that have incorporated Reactome data or tools into their research), as well as news items, the Twitter feed, curation statistics from the most recent release, and information about the project.
  Scrolling farther down on the home page (Fig. 3) reveals a “help” panel, with buttons linking to guides for users and developers, a button linking to information on citing Reactome and a “Contact Us” button, for users requiring help. Below the help panel is a panel for API and data access, including the Content and Analysis Services, the icon library and the graph database.

**Figure 4:**
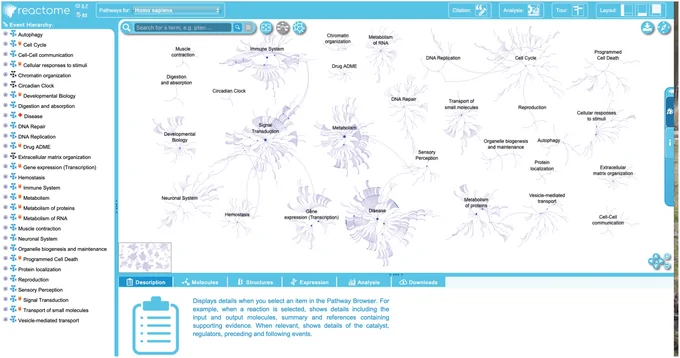
The Pathway Browser, including the event hierarchy (left panel), the details panel (bottom) and the visualization panel displaying the “Pathway Overview” view.

2 To begin exploring the curated Reactome pathways, click on the “Pathway Browser” button on the home page. This will load the page shown in Figure 4.
  The Reactome Pathway Browser consists of four key elements:
  1. *The* ***header bar*,** *at the top of the page. This has the Reactome logo, which returns users to the home page when clicked. Next to this is a species selector, with a drop‐down list of species. Selecting an organism from the species selector will refresh the pathway browser with the inferred pathway diagram from the selected model organism if it is conserved. Reactome data is human‐centric. Data for other species is inferred from human pathways and pathway steps may be missing for other organisms if they are not identified by the inference process. The “Analysis” button provides access to the interactive tools associated with the pathway diagrams, described below in* *Basic Protocols* *4*, *5*, *6* *, and* *7* *. Clicking the “Tour” button in the header opens a brief video tutorial on the key Reactome website functions, while selecting one of the layout buttons in the top right of the header bar allows users to personalize the Web site panels that are displayed to optimize viewing*.
  2. *The* ***pathway hierarchy panel*,** *occupying the vertical rectangle on the far left of the screen, provides a scrolling display of all Reactome pathways in a hierarchy. The plus (“+”) symbol indicates that there are subheadings underneath the pathway headings. Clicking on a plus (“+”) symbol will expand the topic to show its subsections. The subpathways and reactions within each pathway can be hidden by clicking on the minus (“–”) symbol to the left of the pathway name. Next to the plus/minus signs is a small pathway icon in blue or black, indicating the presence or absence of an “Enhanced High Level Diagram” (EHLD, see below) associated with that pathway. A red “N” or “U” next to a pathway name indicates that the pathway is new or has been updated since the last release, respectively. A red cross next to a pathway name indicates that the pathway contains disease annotations*.
  3. *The* ***visualization panel,*** *to the right of the hierarchy panel, displays an interactive pathway diagram that can be panned and zoomed in Google Map style. The visualization panel is synced with the pathway hierarchy on the left, such that selecting pathways or subpathways in the hierarchy will change what is displayed in the visualization panel. There are three primary views that can be displayed in this panel. The first view, “Pathway Overview”, displays the entire pathway hierarchy as interconnected nodes, with nodes representing pathways and edges representing relationships. If a user selects a pathway in the hierarchy or in the graphical display, the corresponding node is outlined in orange. The second view, “Enhanced High Level Diagram (EHLD)”, where available, displays a textbook style interactive illustration of a user‐selected pathway (* *Sidiropoulos et al.*, *2017* *). The third view, “Entity Level View” (ELV), displays the reaction‐level molecular details of the user‐selected pathway. The ELV pathway diagrams apply the conventions of the Systems Biology Graphical Notation (SBGN) format (* *Le Novère et al.*, *2009* *) to distinguish the molecules and reactions by shape and cellular location, providing a dynamic framework for pathway visualization and data analysis. Users can toggle between the Pathway Overview and the ELV views by clicking the second of three blue icons beside the search bar at the top left of the visualization panel. EHLDs, where available, appear as a thumbnail in the bottom left of the visualization panel, and can be accessed by clicking the pathway name in the pathway hierarchy at the left of the visualization panel. An alternate view of the entire pathway hierarchy can be accessed by clicking the third blue icon to the right of the search bar from the Pathway Overview view. This opens a separate window and displays the Reactome pathways as a Voronoi diagram, with sizes of pathway clusters proportional to the number of events the pathway contains (Jassal et al.*, *2020* *). To move from the Voronoi diagram back to the Pathway Browser, click and hold on a pathway name within the Voronoi image*.
  At the top left of the visualization panel is a search bar, featuring results grouping and filtering, hit highlighting and text auto‐completion (Fabregat et al., 2016). The visualization panel also contains an icon (top right, first blue icon) that allows export of the pathway visualization in several different formats, a “compass” icon that provides a pathway overview key (top right, second blue icon) and an expandable panel on the right side which provides information and allows users to customize color preferences.
  At the bottom right of the visualization panel are navigation arrows and zoom features. Users can also zoom using the mouse wheel and can click and drag the diagram. The thumbnail image, in the lower left corner of the visualization panel, can be used to navigate quickly to a region of interest in the pathway diagram.
  4. *The* ***details panel*** *is located below the visualization panel, and its contents change in sync with user selections in the visualization panel or the pathway hierarchy. The details panel has 6 tabs, each of which contains a general description of what will be displayed in that panel once an event or entity is selected. The “Description” tab displays molecular details related to the selected event or entity, including inputs and outputs of reactions, catalysts, regulators, preceding, and following events, linkouts to other databases with entity information, etc. This tab also displays event summations, literature references, and editorial information. The “Molecules” tab shows downloadable details of the molecules (proteins, small molecules/chemical compounds, nucleic acid sequences) involved in the selected event. The “Structures” tab shows reaction details from Rhea (* *Bansal et al.*, *2022* *) or structural information from ChEBI (* *Hastings et al.*, *2016* *) or PDBe (* *Armstrong et al.*, *2020* *), as appropriate. The “Expression” tab displays gene expression information from Gene Expression Atlas for genes corresponding to the selected items. The “Analysis” tab displays the pathway‐specific results generated by the Reactome analysis tools, and finally, the “Download” tab allows users to download the selected pathway in several different formats*.

**Figure 5:**
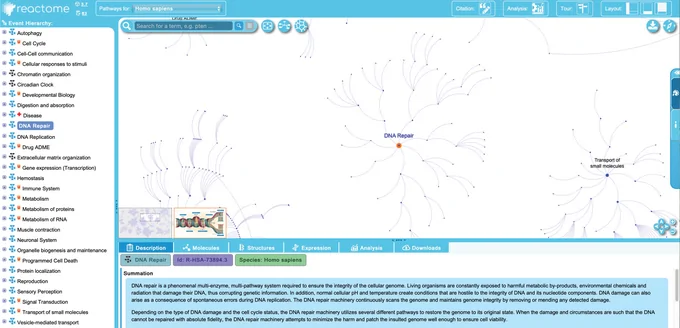
High‐level view of the DNA Repair pathway in the Pathway Browser, showing pathway‐level summation in the details panel, a zoomed‐in view of the Pathway Overview and a thumbnail of the textbook‐style illustration in the visualization panel.

3 This protocol will illustrate features of the Reactome pathway browser by exploring the events contained within the “DNA Repair” pathway. To begin, click on the “DNA Repair” pathway title in the pathway hierarchy at right.
  In response to this selection, the visualization panel zooms in on the DNA Repair node in the Pathway Overview (see Fig. 5) and brings up the pathway level information in the “Description” tab of the details panel, including pathway level summation, editorial attributes (authors and reviewers), literature references, GO Biological Process where appropriate, and cellular compartment. Each of these attributes is expandable: clicking on the plus (“+”) symbol on the right reveals further details, including linkouts to PubMed, ORCID, GO, or other cross‐referenced resources. The Description tab of the details panel also contains a stable identifier for the event displayed, including the three letter species code (HSA for Homo sapiens is the default unless species is changed). Scrolling down in the details panel to the section labeled “View computationally predicted event in” reveals a species selector bar. Reactome's manual annotations are human‐focused but are computationally extended to other species based on protein conservation as described under “Computationally inferred events” in the “Docs” section of the Web site. Selecting a different species from the species selector bar will update the events and information displayed in the visualization and details panel for the selected species. Not that the species may also be changed using the drop‐down menu to the right of the Reactome logo in the header of the pathway browser.
  Other tabs in the details panel are also updated in response to the selection of “DNA Repair” pathway in the hierarchy (note that the “Structures” tab is not populated with data when a pathway‐level event is selected in the hierarchy, and the “Analysis” tab is only populated once an analysis has been performed). The “Molecules” tab is updated to provide information on all the chemical compounds (55 in DNA Repair pathway), proteins (315) drugs (28), sequences (0) or other entities (0) contained in the pathway, and the total number of molecules is displayed as in a red bubble at the top of the “Molecules” header (398). Inside the “Molecules” panel, expandable sections provided detailed information on each of the entities contained in the event linked out to the appropriate reference database. This information is downloadable in full or in part from within the “Molecules” tab. The “Expression” tab displays expression data from Gene Expression Atlas for each of the genes contained within the pathway, and the “Download” tab has pathway reports and diagrams for the selected pathway available for download in a variety of formats.

**Figure 6:**
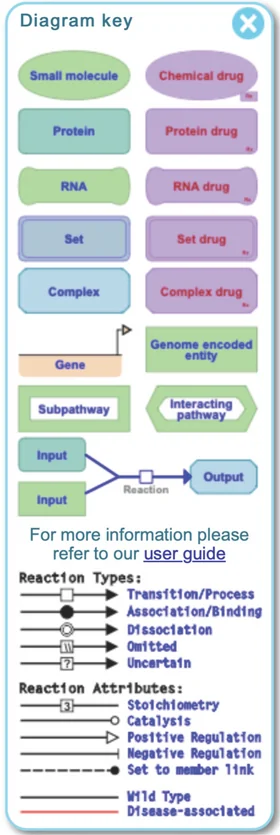
A key to the SGBN‐based icons used in the molecular level events in Reactome.

4 Double click on the “DNA Repair” pathway title in the hierarchy, or double click on the corresponding node in the visualization panel.
  Double clicking on the DNA Repair pathway title in the hierarchy opens the interactive EHLD for this pathway (users know an EHLD is available because the pathway icon between the plus sign and the pathway name in the hierarchy is blue rather than black; pathways with black icons do not have EHLDs but may have static, non‐interactive illustrations).
5 Click on the plus (“+”) symbol beside “DNA Repair” in the hierarchy to reveal the subpathways.
  Users can navigate to any of the 7 subpathways of DNA Repair by clicking on the plus (“+”) symbol beside DNA Repair in the hierarchy or by clicking on the subpathway name in the EHLD. Clicking on a subpathway name either in the hierarchy or from the EHLD will either open another EHLD, if the selected subpathway itself contains multiple subpathways with ELV diagrams (as is the case for the Base Excision Repair subpathway) or will open an ELV pathway as is the case for the other 6 subpathways of DNA Repair.
6 Click on the “Nucleotide Excision Repair” pathway either in the hierarchy or from within the “DNA Repair” EHLD.
  An ELV diagram containing reactions curated at the level of molecular participants appears in the visualization panel, and the details panel updates to reflect information appropriate for this pathway. Note that the total molecules displayed in the “Molecules” tab for this pathway is 119 (9 “Chemical Compounds”, 110 “Proteins”), fewer than the parent DNA Repair pathway, as expected.
  ELV pathways open at a fully zoomed out level. Diagram zoom level is controlled either by a mouse or with the zoom icons in the bottom right of the visualization panel. Diagrams can be easily recentered to the fully zoomed out view by clicking on the icon to the immediate right of the search bar at the top of the visualization panel.
  Depending on the diagram size, commonly occurring reaction participants such as H_2_O or ATP may not be displayed in the diagram at the fully zoomed out level. As the user zooms in, these entities are dynamically added to the pathway diagram.
  Nucleotide Excision Repair has two subpathways, both laid out in the same ELV: “Global Genomic NER (GG‐NER)” and “Transcription‐coupled NER (TC‐NER)”. These subpathway names are displayed in the zoomed‐out view of the ELV and are contained by colored subpathway boundary boxes. Clicking on either of the NER subpathway names in the hierarchy highlights the events encompassed by that subpathway in the visualization panel in blue, while hovering over a pathway name highlights the corresponding events in yellow.
  The pathway diagram uses the conventions of the Systems Biology Graphical Notation (SBGN) format to distinguish the molecules and reactions by shape and cellular location, to provide a dynamic framework for pathway visualization and data analysis. A key to the shapes used in the ELV diagrams is available by clicking on the compass arrow at the top right of the visualization panel; doing so reveals the key shown in Figure 6.

**Figure 7:**
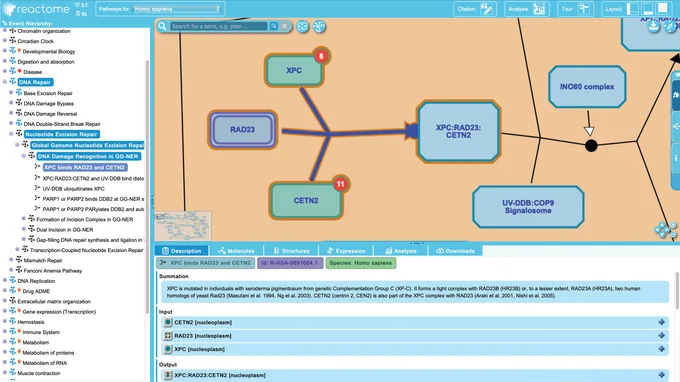
Selection of a particular reaction (“XPC binds RAD23 and CETN2” in the “DNA damage and recognition in the GG‐NER” pathway) updates the Pathway Browser appropriately.

7 Continue to drill down into the hierarchy to reach reaction level events as follows: Click on the pathway title “Global Genomic Nucleotide Excision Repair”. Note that the details panel shows that this pathway contains 92 of the 119 molecules present in the NER pathway (8/9 “Chemical Compounds” and 84/110 “Proteins”). Expand this subpathway in the hierarchy by clicking on the adjacent plus sign to reveal the four subpathways (“DNA Damage Recognition in GG‐NER”, “Formation of Incision Complex…”, etc.). Continue to expand the hierarchy by clicking the “DNA Damage Recognition in GG‐NER” pathway in the hierarchy to reveal the five molecular level events contained within, noticing that at each subsequent pathway level the fraction of molecules represented is adjusted relative to the parent NER pathway.
8 Click on “XPC binds RAD23 and CETN2”, the first reaction in the “DNA Damage Recognition in GG‐NER” pathway, as shown in Figure 7.
  Clicking a reaction in the pathway hierarchy will cause the reaction name and the name of the subpathway(s) and parent pathways to be highlighted, as seen in Figure 7, above. The visualization panel will recenter on the selected reaction and the reaction will be highlighted in blue. Furthermore, the description tab of the “Details” panel will update to show particulars of the selected reaction, which will include some or all of the following, where appropriate: inputs, outputs, catalysts and positive and negative regulators, preceding event, and “inferred from” reaction.
  In the case of the “XPC binds RAD23 and CETN2” reaction, there are three inputs, one output, no catalyst or regulators, no preceding event, and no “inferred from” reaction. The inputs to the reaction are the proteins XPC and CETN2 and a set of RAD23 proteins. Reactome sets are groups of molecules that are known or predicted to function in the same way in a given reaction and may either be “member” sets, where each participant is known to have the indicated role, or “candidate” sets, where some participants are verified members, and others are candidates based on sequence or structural similarity. Here the RAD23 set is a “candidate” set, with RAD23B a verified member and RAD23A a candidate. The output of this reaction is the complex formed by the binding of the three inputs (XPC, the RAD23 set, and CETN2).
  Clicking on the “XPC binds RAD23 and CETN2” reaction simultaneously updates information in the other details panel tabs:
  ‐The red bubble on the “Molecules” header now indicates that the reaction contains 4/119 molecules from the Nucleotide Excision Repair pathway, all of them proteins, and the “Molecules” panel is updated with information on these four proteins.
  ‐The “Structures” header has been decorated with a red bubble indicating the fraction of the molecules in the reaction for which structural information is available (here, 4/4), and the panel provides linkouts to those structures in Protein Data Bank. The expression for these four proteins is displayed in the “Expression” tab.
  ‐The “Analysis” tab continues to be blank unless an analysis has been performed.
  ‐The “Download” tab: note that although a reaction is selected in hierarchy, the download available from this tab remains the immediate parent ELV pathway (here the “Nucleotide Excision Repair” subpathway of the top‐level “DNA Repair”).
  In the context of the “DNA Damage and Recognition in GG‐NER” pathway, the reaction “XPC binds RAD23 and CETN2” is the first in a series of connected reactions, in which the output of one reaction is the input to the next reaction. Because there are no annotated reactions that occur prior to the binding of RAD23 and CETN2 to XPC, there is no indication of a “preceding event” in the “Description” tab of the details panel for this reaction. If the following reaction in the hierarchy (“XPC:RAD23:CETN2 and UV‐DDB bind distorted dsDNA site”) is selected, the “Description” tab of the details panel is updated with reaction‐specific information. In particular, a new field describing the positive regulator (INO80 complex) is added, as is the new field “Preceding Event”, which lists the “XPC binds RAD23 and CETN2[Homo sapiens]” reaction described above. Clicking on the plus (‘+’) symbol to the right of this “preceding event” reaction name expands this field to reveal the summation and literature references associated with the reaction. This allows users to put the current reaction into a more complete biological context.
  The relationship between the “levels” of the pathway hierarchy on the one hand and the “Preceding event(s)” links, on the other hand, may not be immediately clear. The nested levels of the pathway hierarchy reflect levels of abstraction in the conceptual organization of pathways. As one moves deeper into the hierarchy, the contents of the pathway diagram become more and more specific and move closer to the biochemical reaction level. The “Preceding event(s)” link only appears at the reaction level.
  Reactions in Reactome are human‐centric. Wherever possible, the molecular events that are depicted in Reactome are supported by direct experiments that make use of human cells, tissues, protein, or other entities. This evidence is cited in the literature references associated with the reaction. Often, however, knowledge of human biology is derived indirectly from work using model organisms. If the direct experimental evidence supporting a given reaction is model organism‐based, an “inferring” reaction is created using the molecules from the relevant species, and a corresponding human reaction is inferred from it. Human reactions that are inferred in this way are indicated with a double arrow adjacent to the reaction title in the hierarchy, and by the presence of an “inferred from another species” field in the “Description” tab of the details panel. Expanding the field by clicking on the plus (‘+’) symbol at the right reveals the summation and references for the experiment(s) in the other species. “Inferred from” reactions may also be marked as chimeric if the experiment being cited as evidence contains molecules (proteins, nucleic acids, etc.) from multiple species.

**Figure 8:**
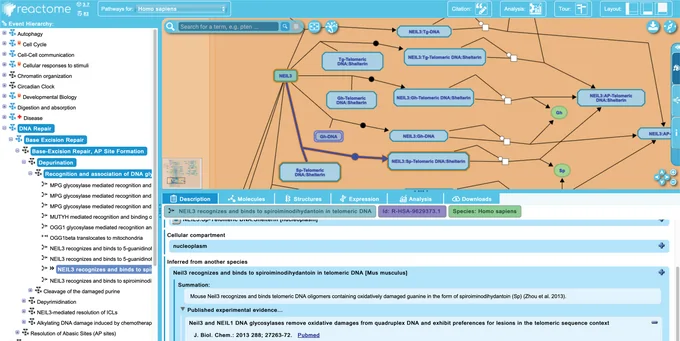
The reaction “NEIL3 recognizes and binds to spiroiminodihydatoin in telomeric DNA” is inferred from a corresponding reaction in *Mus musculus*, as evidenced by the double arrowhead beside the event name in the hierarchy.

9 There are no inferred reactions within the “Nucleotide Excision Repair” subpathway. To see an example in the “Base Excision Repair” pathway, expand the “Base Excision Repair” hierarchy to reveal the subpathways “Base‐Excision Repair, AP Site Formation”, and continue to unfurl its child pathway “Depurination”, and **its** child pathway “Recognition and association of DNA glycosylase with site containing an affected purine”.
  This pathway contains 10 reactions, the ninth of which is “NEIL3 recognizes and binds to spiroiminodihydantoin in telomeric DNA”, with the inferred double arrow indicator beside its event name in the hierarchy. The “Description” tab of the details panel contains an “Inferred from another species” field, listing the corresponding reaction from Mus musculus, along with its summation and literature references, as shown in Figure 8.

**Figure 9:**
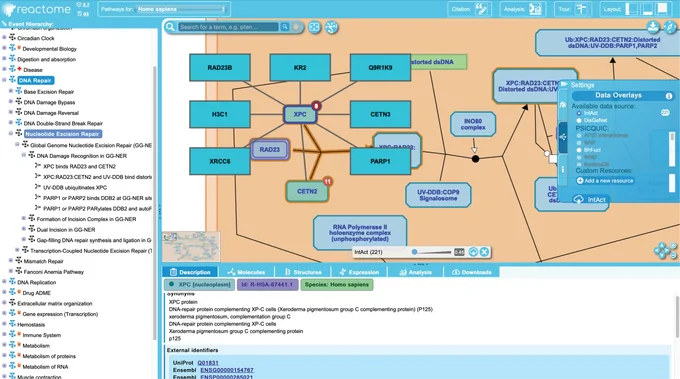
Proteins that interact with XPC as described in the IntAct database are displayed as a halo around XPC in the visualization panel.

10 In addition to the reaction level information described above (summations, literature references, editorial attributes, etc.), Reactome also provides detailed information about each of the entities involved in a reaction. To explore this, return to the first reaction of the “DNA Damage Recognition in GG‐NER pathway”, “XPC binds RAD23 and CETN2”. Clicking on any of the inputs or outputs of the reaction (or regulators and catalysts where applicable) updates the “Description” tab of the details panel with information and linkouts for the corresponding molecule. In the “XPC binds RAD23 and CETN2” reaction, click on the “XPC” entity in the pathway diagram.
  This will update the Details panel to display information about the protein, including synonyms, cellular compartment (linked out to the GO ontology), external identifiers to resources such as UniProtKB, Ensembl, GeneCards, HMDB Protein, HPA, OpenTargets, Orphanet, PDB, PRO, Pharos, and RefSeq and a selector bar to view the entity in other species. Small molecule inputs or outputs (none in this reaction) are similarly linked to appropriate reference databases and provided with synonyms and cellular compartments in the “Description” tab of the details panel. Small molecules are given species‐agnostic identifiers beginning with R‐ALL.
  Note that in the “XPC binds RAD23 and CETN2” reaction, the XPC and CETN2 icons in the visualization panel are decorated with red circles on the upper right corner. This mark indicates the number of interacting proteins in the IntAct database for that entity. Clicking on the red icon displays the interacting proteins in a halo around the pathway entity, as shown for XPC in Figure 9; clicking on an interactor takes the user to the UniProtKB record for that protein. In cases where there are too many interactors to display in the context of the pathway diagram, only the top 18 interactors are shown. Users can customize the confidence level for these interactors with the sliding scale at the bottom of the visualization panel; this toolbar also allows users to download all pathway interactors as a CSV file.
  Although the interacting protein overlay by default is set to IntAct, users can change the referring database by opening the panel on the right side of the visualization panel as shown in Figure 9 and selecting one of the other highlighted resources from the middle “Interactors” tab. These interactors are loaded on an on‐demand basis through PSICQUIC. Note that at this time, interactors can only be displayed for individual pathway protein entities and not for complexes or molecule sets.
  Also note that zooming in on XPC or other proteins within the visualization panel reveals within the pathway icon the UniProtKB identifier and, if available, an associated structure from PDB.

**Figure 10:**
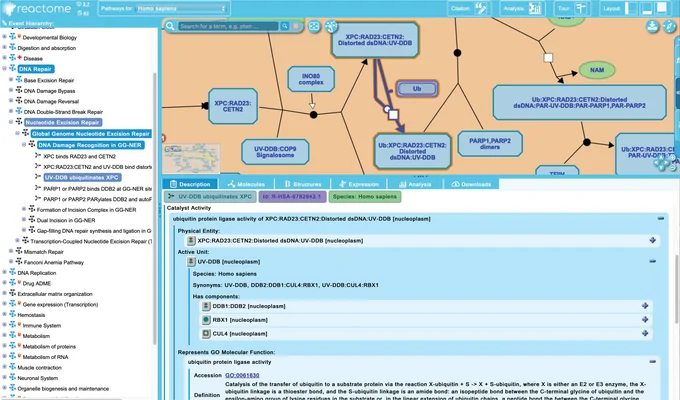
The details of a Reactome catalyst are shown in the context of the reaction “UV‐DDB ubiquitinates XPC”.

11 Reactome provides information about the subunits of a complex, as well as the larger ensembles of proteins that a complex participates in. In this example, from the “XPC binds RAD23 and CETN2” page, click on the “XPC:RAD23:CETN2” entity in the pathway diagram. This will update the Details panel to display information about this complex.
  The “Description” tab of the details panel will now include a new field “Components”, in which each of the constituents of the complex are listed, each with an expandable window that links to further information about that entity (synonyms, compartment, reference entities, external identifiers, etc. as outlined above for XPC; in addition, clicking on the icon to the left of the component name—here, the green protein circle for XPC also reveals a window with entity‐specific information).
  In addition, the “Description” tab of the details panel now includes a “Produced by” and a “Consumed by” field, listing events across Reactome as a whole in which the complex is either an output or an input, respectively. These fields are expandable [plus (‘+’) symbol at right] to reveal summation and literature references for the reaction‐like‐event in question. Clicking on the reaction icon to the left of the “Produced by” or “Consumed by” reaction titles will highlight the reaction node and recenter the visualization panel on the corresponding reaction. Note that this may move the user to a different pathway diagram.
  More detailed information is also provided about the components of sets; to see this click on the RAD23 set that is an input to the “XPC binds RAD23 and CETN2” reaction. Note, however, that sets are not associated with “Produced by” or “Consumed by” fields as complexes are.
12 To explore the information Reactome provides about catalysts, click on the third reaction in the “DNA Damage Recognition in GG‐NER” pathway, “UV‐DDB ubiquitinates XPC”. Catalysts are shown regulating the reaction node by virtue of an edge ending in a circle (see Fig. 10). Catalysts may either be independent of other reaction participants, or, as in this case, may be one of the reaction inputs. Reflecting this dual role, the “XPC:RAD23:CETN2:Distorted ds DNA:UV‐DDB” complex has both a reaction edge and a catalyst edge associated with it.
  In addition to the fields described above for reactions without catalysts, the “Description” tab of the details panel for an enzyme‐catalyzed reaction also contains the following information about the catalyst (Fig. 10):
  ‐Physical Entity: whichever molecule in the pathway diagram is associated with the catalyst activity. This may be a single protein, a set of proteins, or a complex (here the complex “XPC:RAD23:CETN2:Distorted ds DNA:UV‐DDB”).
  ‐Active Unit: in cases such as this one where a complex is the catalyst, the specific component that contributes the catalytic activity is identified. Here, the active unit is the UV‐DDB subcomplex consisting of DDB1 and 2, RBX1 and CUL4.
  ‐Molecular Function: the most appropriate term is taken from (and linked out to) the GO. The catalyst name is a concatenation of the GO Molecular Function and the name of the Physical entity.

**Figure 11:**
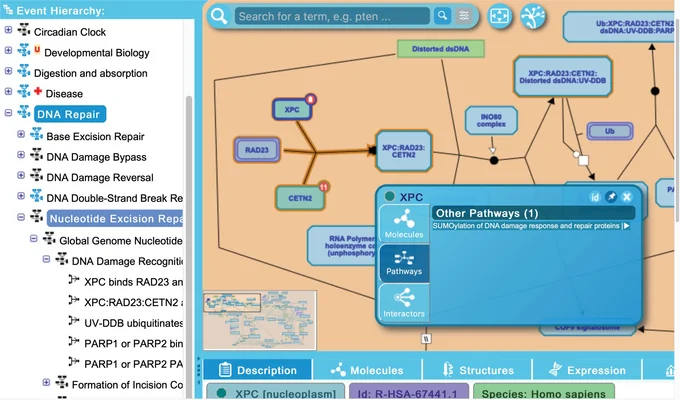
The Contextual Information Panel (CIP) displays information about a given pathway entity, including constituent molecules, other Reactome pathways where that entity occurs, and interactors.

13 Reactome provides inter‐pathway connections for physical entities contained within a given pathway diagram. Hovering over any entity in the visualization panel reveals an arrowhead at the right side of the entity icon. Clicking on this arrowhead reveals an interactive information panel (Contextual Information Panel, CIP) with three tabs (see Fig. 11): “Molecules”, “Pathways”, and “Interactors”. Similar to the descriptions above, the “Molecules” tab provides the components of the selected entity, and the “Interactors” tab provides a table listing the interacting proteins along with scores and evidence (note that display name of components or interactors can be toggled between common name and reference identifier by clicking on the small “id” button at the top right of the interactive panel; clicking on the pin icon locks the interactive panel to the pathway diagram; to close the panel, click on the “x” icon).
  To explore the “Pathways” tab, click on the arrowhead revealed by hovering above the XPC protein input in the first reaction of the “DNA Damage Recognition in GG‐NER” subpathway, “XPC binds RAD23 and CETN2”.
  The “Pathways” tab lists other Reactome pathways in which the selected entity takes part; here the only other pathway in which XPC has an annotated function in Reactome is the “SUMOylation of DNA damage response and repair proteins” pathway. Clicking on the pathway name within the interactive panel moves the user to the new pathway in which that entity participates.
  This connection between pathways mediated by shared participants highlights potentially unexpected linkages between disparate areas of biology and illustrates the power of Reactome to bridge the domains.

## Basic Protocol 2 EXPLORING REACTOME ANNOTATIONS OF DISEASE AND DRUGS

In addition to normal human biology, Reactome annotates abnormal or pathological events arising from genetic mutation or interaction with an infectious agent in a separate top‐level pathway called “Disease”. Reactome disease pathways are designated with a red “+” symbol to the left of the pathway name and include cancer, metabolic, immune, and infectious diseases, among others. Where possible, Reactome disease pathways also include the interaction of relevant therapeutic drugs.

Consistent with Reactome's pathway‐centric view, disease events (with the exception of infectious processes) are annotated as changes to normal molecular level reactions and are displayed in the context of the relevant non‐disease pathway background. As a result, there is no single diagram representing a given disease (e.g., bladder cancer or diabetes) but rather individual events that are perturbed in the course of that disease are labeled with the appropriate disease tag and displayed as overlays to normal pathway events. Events with the same disease tag may, therefore, be distributed across multiple normal pathways and pathway diagrams. Infectious diseases represent novel events that do not have a corresponding normal state and have their own pathway diagrams.

Reactome's disease and drug annotations will be explored using the disease pathways “Signaling by ERBB2 in Cancer” and “SARS‐CoV‐2 Infection”. This module will highlight where and how the disease pathway annotations diverge from those of normal pathways; many of the key annotation features, however, are functionally equivalent and these will not be detailed here.

### Necessary Resources

#### Hardware

Computer capable of supporting a Web browser and an Internet connection

##### Software

Any modern Web browser such as Firefox, Safari, and Chrome will work to display Reactome Web pages

**Figure 12:**
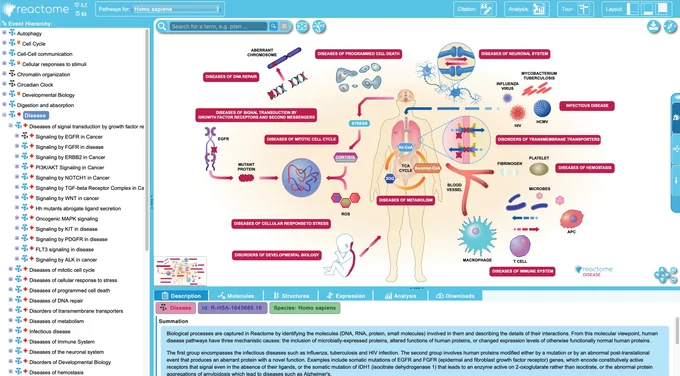
The textbook‐style illustration and the pathway summation for the top‐level Disease pathway.

1 To begin exploring Reactome's disease and drug annotations, point the browser to the Reactome home page at https://reactome.org.
2 Click on the “Pathway Browser” button on the home page and unfurl the events under the “Disease” top level pathway in the hierarchy by clicking on the “+” symbol to the left of the pathway name.
  Notice that subpathways are grouped by general biological processes (“Diseases of signal transduction by growth factor receptors and second messengers”, “Diseases of metabolism”, “Infectious diseases”, etc.) rather than by specific diseases (diabetes, bladder cancer, etc.). These groupings generally mirror the pathway groupings of the normal event hierarchy (See Fig. 12).

**Figure 13:**
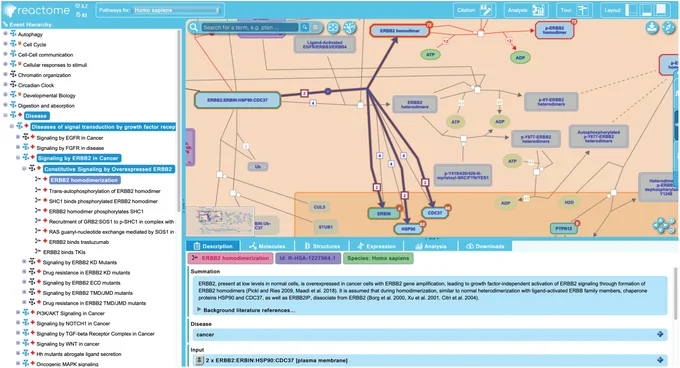
A gain‐of‐function reaction in the “Constitutive Signaling by Overexpressed ERBB2” pathway.

**Figure 14:**
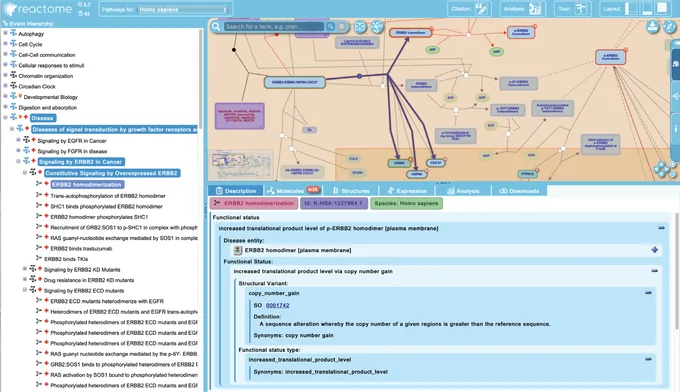
The details panel identifies the “Functional Status” of the disease entity (here, p‐ERBB2 homodimer), describing the underlying genetic changes that result in abnormal molecular behavior and disease outcomes.

3 Continue to unfurl the disease hierarchy, first expanding the “Diseases of signal transduction by growth factor receptors and second messengers” pathway, and then the child of that subpathway “Signaling by ERBB2 in Cancer”. This is an EHLD‐level pathway with 6 subpathways.
  Notice that in the “Description” tab of the details panel for this pathway, there is a new field “Disease”, with “cancer” as the entry. Expanding this field with the “+” at the right reveals a disease definition and synonyms along with a hyperlinked identifier for the disease that refers back to the corresponding page in the Human Disease Ontology at the OLS (Schriml et al., 2019). DOIDs are applied to all disease entities and events. In the case of disease entities, such as proteins that arise as the result of genetic mutation, the entity may be labeled with as many separate DOIDs as there is evidence to support; in the case of sets of disease entities AND in the case of disease events, only DOIDs that are applicable to ALL the contained entities/events are applied. For this reason, pathway‐level DOIDs are often quite general (“cancer” in this case). Drilling down into the hierarchy usually corresponds to more and more specific disease terms.
4 Click on the first subpathway “Constitutive Signaling by Overexpressed ERBB2”. This opens an ELV level pathway with molecular level reactions laid out.
  Relative to a wild‐type pathway, ELV‐level disease pathways have two new fields in the “Description” tab of the details panel, “Disease” as described above and “Normal pathway”. The “Normal Pathway” field lists the non‐disease pathway from the normal hierarchy upon which the disease events are overlaid ‐ in this case “Signaling by ERBB2”. Expanding the panel reveals the summation for the wild‐type pathway, while clicking on the pathway icon to the left of the normal pathway name in the details panel moves the user to the pathway diagram for that normal pathway. To return to the disease ELV from the normal pathway, click on the browser's back button. Notice that the events of the normal pathway, while visible in the disease ELV, are not clickable or interactive. To demonstrate this, hover over or try to click any of the greyed‐out reaction lines or entities from the normal pathway.
  Disease entities and disease reaction lines are highlighted in the disease ELV in red, while drugs and drug‐containing entities are colored purple and have a small R_x_ in the bottom right of the icon. All disease and drug entities and events displayed in the ELV are fully interactive as expected.
5 Click on the first reaction of the “Constitutive Signaling by Overexpressed ERBB2” pathway, “ERBB2 homodimerization”.
  This reaction has as input a complex of ERBB2 with the protein ERBIN and the chaperone proteins HSP90 and CDC37; during the reaction, 2 such complexes (note the stoichiometry displayed on the reaction line) associate, the chaperone proteins and ERBIN are released and an ERBB2 homodimer is formed (see Fig. 13).
  Notice that the input complex (“ERBB2:ERBIN”HSP90:CDC37″) and the outputs ERBIN, HSP90, and CDC37 are not colored red. These are all genetically normal proteins or protein complexes, are not labeled with a disease tag and consequently are not colored red. In contrast, the output (ERBB2 homodimer) is colored red, and is associated with a disease tag (although this annotation is not currently displayed in the “Description” section of the details panel when the ERBB2 homodimer is selected in the pathway diagram). In this example, although all the participating input entities are genetically normal, the process is not: disease‐associated amplification of the ERBB2 gene leads to protein overexpression. This allows the receptor tyrosine kinase to homodimerize in the absence of ligand, something that does not occur under normal conditions (the corresponding complex under normal conditions would be a heterodimer consisting of a monomer of ERBB2 and a ligand‐activated monomeric member of the ERBB family (EGFR, ERBB3, or ERBB4)). For this reason, the ERBB2 homodimer is a disease entity, is marked with a disease tag and is colored red.
  This reaction represents a gain‐of‐function or novel reaction, in that the protein is carrying out a new role that is not seen in the normal pathway. Infectious disease events are another example of gain‐of‐function events, as by definition the presence of an infectious agent is a new, disease‐associated attribute.
  Although gain‐of‐function disease reactions are displayed in the context of the normal, grayed‐out pathway (and may make use of other components of the normal pathway, as in this case), the actual reaction is generally laid out separately in its own space in the pathway diagram. This is in contrast to loss‐of‐function disease reactions, described below in point 9, which are overlaid directly on top of the corresponding normal reactions. Exceptions to this “separate space for gain‐of‐function reactions” rule are explained below in point 8.
  Disease reactions display a new field in the “Description” tab of the details panel relative to normal events: “Functional Status”. This expandable field identifies the disease physical entity, as well as the underlying genetic structural variation and the functional outcome of that variation, with terms pulled from Sequence types and features ontology (SO; Eilbeck et al., 2005). This is shown in Figure 14.

**Figure 15:**
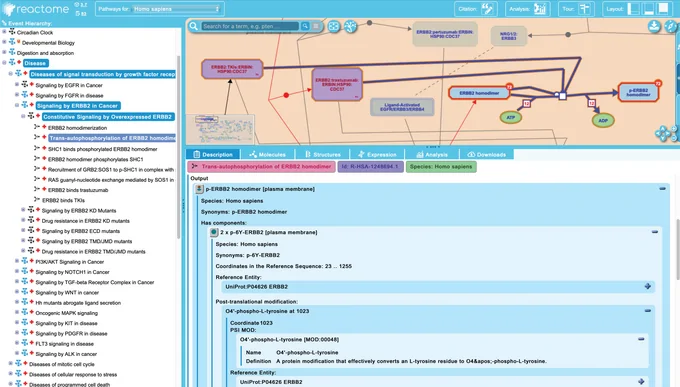
The details panel captures precise information about post‐translational modifications to pathway entities.

6 Click on the next reaction in the “Constitutive Signaling by Overexpressed ERBB2”, “Trans‐autophosphorylation of ERBB2 homodimer”.
  This reaction shows the post‐translational modification of the receptor through autophosphorylation, and is highlighted here not to illustrate a disease‐specific reaction element, but rather as a demonstration of the ability of Reactome to capture detailed post‐translational modifications. Scroll down in the “Description” tab of the details panel for this reaction and unfurl the field for the output, “p‐ERBB2 homodimer”. Notice that this complex consists of two copies of the phosphorylated ERBB2 monomer. Phosphorylations are listed under the “Post‐translational modification” field, which includes the coordinate(s) of amino acid residues from the reference sequence that are modified and details of the specific modifications with terms taken from PSI MOD (see Fig. 15). Notice also that this reaction has two negative regulators, the complexes “ERBB2:TKIs:ERBIN:HSP90:CDC37” and “ERBB2:trastuzumab:ERBIN:HSP90:CDC37”. These therapeutic‐containing complexes are appropriately shaded in purple to reflect the inclusion of drug(s) and will be described in more detail in step 7 of this protocol.

**Figure 16:**
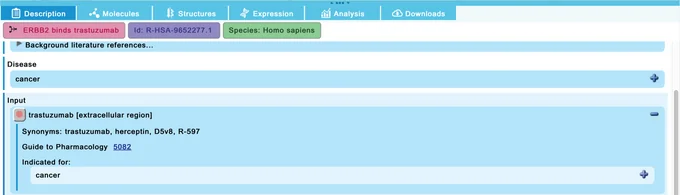
Reactome captures the effect of therapeutics on pathway events where possible and links the therapeutics to appropriate external resources in the details panel.

7 In the “Constitutive Signaling by Overexpressed ERBB2”, click on the reaction “ERBB2 binds trastuzumab”. Scroll down in the “Description” tab of the details panel and expand the field for the “trastuzumab” reaction input.
  Reactome annotates therapeutics in three classes ‐ protein, small molecule, and RNA drugs. Drugs are cross‐referenced to Guide to Pharmacology, ChEBI and/or PubChem where possible and applicable, and this information is displayed in the “Description” tab of the Details panel as shown in Figure 16. Similar information can also be viewed by clicking on the red circle icon beside the trastuzumab name.
  The effect of the drug on the disease pathway is annotated where possible. In this pathway, the complex formed by the binding of the monoclonal antibody Trastuzumab to the ERBB2‐chaperone complex inhibits the ability of the ERBB2 homodimer to autophosphorylate, thus preventing signaling downstream of the abnormally activated receptor. In the case of the “ERBB2 binds TKIs” reaction, Reactome shows the binding of a set of small molecule tyrosine kinase inhibitors (TKIs) to the ERBB2:chaperone complex; this binding inhibits the tyrosine kinase activity of the receptor, similarly preventing autophosphorylation and downstream signaling.

**Figure 17:**
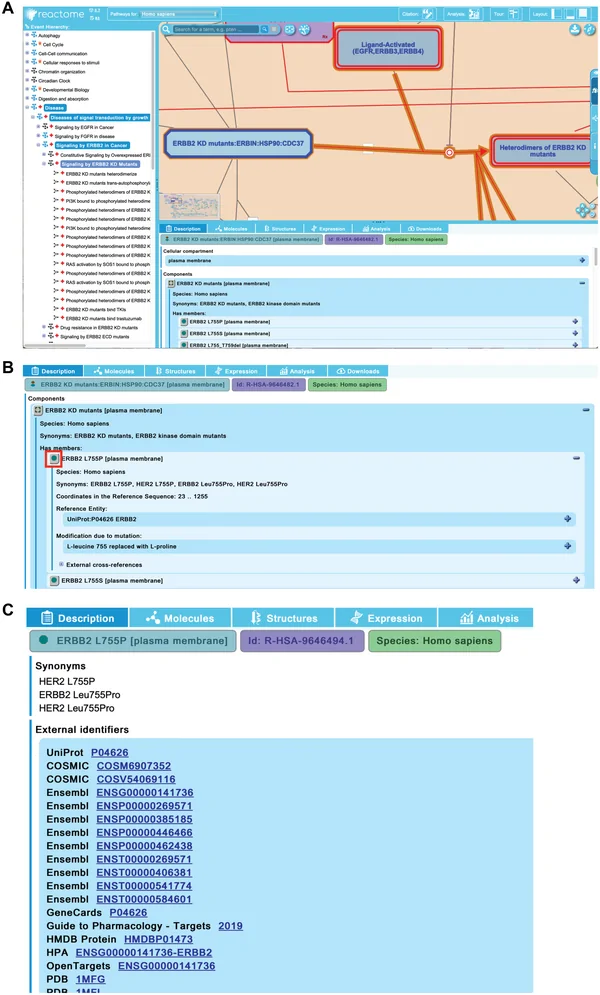
**(A)** A list of the members and candidates of the “ERBB2 KD mutants” set is revealed by clicking on the “+” symbol on the right side of the details panel after the disease complex is selected in the pathway diagram. **(B)** Detailed molecular information about the genetic changes that give rise to mutant ERBB2 L755P is displayed by clicking on the “+” symbol to the right of the variant name in the details panel Linkouts to appropriate databases and ontologies are provided. **(C)** Further cross‐references are available by clicking on the green circle to the left of the disease entity name.

8 Reactome captures detailed molecular information about individual proteins that are implicated in abnormal biochemical reactions in disease. To explore this, unfurl the second pathway of “Signaling by ERBB2 in Cancer”, “Signaling by ERBB2 KD Mutants”, and click on the first reaction “ERBB2 KD mutants heterodimerize”.
  This pathway describes ERBB2 proteins with mutations in the kinase domain (KD) that increase the catalytic activity of the enzyme, resulting in elevated autophosphorylation and downstream signaling. In this case, the mutant proteins are performing the same biochemical role as their normal counterparts but at an elevated rate or efficiency. In cases like this, the disease reactions are overlaid directly on top of the corresponding normal ones in the diagram, unlike in the case of novel functions described in step 5, above, for overexpressed ERBB2.
  To see the details of the ERBB2 mutants annotated in this reaction, click on the complex “ERBB2 KD mutants:ERBIN:HSP90:CDC37” in the pathway diagram. In the “Description” tab of the details panel, select the set “ERBB2 KD mutants” to reveal the list of member and candidate proteins (Fig. 17A). Expand the field for the first member, “ERBB2 L775P” with the ‘+’ on the right. This reveals detailed information about the protein including the genetic alteration (here L‐leucine 755 replaced with L‐proline) (See Fig. 17B). Clicking on the green circle beside the “ERBB2 L755P” name (indicated with the red square in Fig. 17B) reveals further information including linkouts to COSMIC (or OMIM, or ClinGen, where appropriate), as well as the literature reference specific for that mutant (scroll down to bottom of the panel to see this) (Fig. 17C).

**Figure 18:**
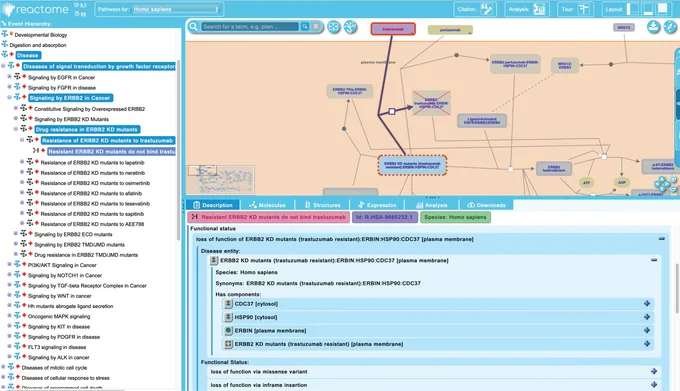
Loss‐of‐function reaction “Resistant ERBB2 KD mutants do no bind trastuzumab” is shown with the loss‐of‐function entity bordered by a broken red line and the output of the failed reaction crossed out.

9 Reactome also annotates loss‐of‐function events, where a protein has lost all or most of its normal functional activity. To explore this, unfurl the third subpathway of “Signaling by ERBB2 in Cancer”, “Drug Resistance in ERBB2 KD mutants”. This reveals a further 8 subpathways, each describing the resistance of sets of ERBB2 KD mutants to 8 different drugs. Select the first pathway, “Resistance of ERBB2 mutants to trastuzumab” and unfurl that pathway to reveal the single “failed reaction”, “Resistant ERBB2 KD mutants do not bind trastuzumab” (Fig. 18).
  In a loss‐of‐function event, the relevant disease entity (here the complex “ERBB2 KD mutants (trastuzumab resistant):ERBIN:HSP90:CDC37”) is outlined with a dashed red line, and products that are no longer made are greyed out with a superimposed red “X”. These loss‐of‐function events represent “stop points” in the pathway and are overlaid directly on top of the corresponding normal event in the pathway diagram.
  The “Description” tab of the details panel provides “Functional Status” information for the disease entity and unfurling the complex to reveal the member and candidate proteins will provide access to detailed mutational and linkout information as described above for gain‐of‐function mutants.

**Figure 19:**
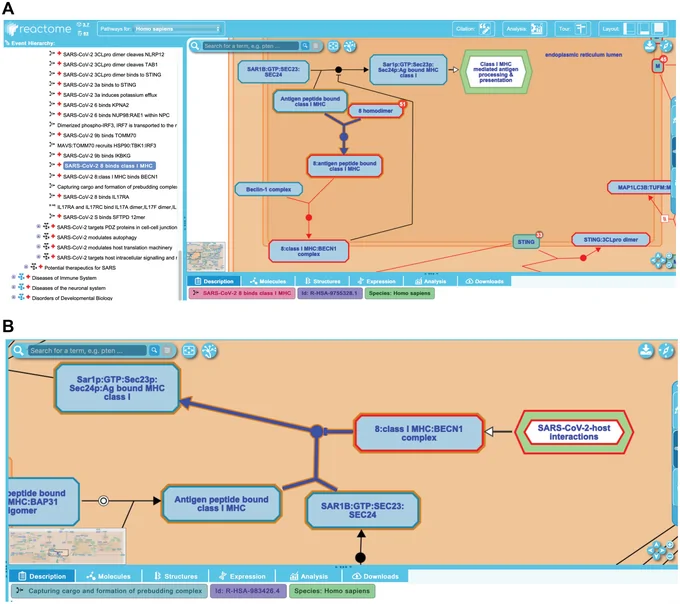
**(A)** Infectious diseases and processes are novel events with no normal counterparts. They are laid out in their own diagrams and are shown interacting with and modulating the function of normal entities and events, as shown here for the SARS‐CoV‐2 subpathway “SARS‐CoV‐2 host interactions”. **(B)** Encapsulated pathway icons like “SARS‐CoV‐2 host interactions” shown here provide connections between pathways that share entities or events.

10 Infectious processes are, by definition, novel events that do not occur in the absence of the initiating pathogen. As such, these are represented in their own diagrams with no corresponding normal pathway. In addition to annotating the infection process itself, Reactome also shows how these infectious agents modulate normal biological processes.
  To explore this, unfurl the “Infectious disease” child of “Diseases”, then continue to unfurl “SARS‐CoV Infections”, “SARS‐CoV‐2 Infection”, and “SARS‐CoV‐2‐host interactions”. This reveals an ELV pathway diagram displaying five subpathways. Expand the subpathway “SARS‐CoV‐2 activates/modulates innate and adaptive immune responses” and click on the reaction “SARS‐CoV‐2 8 binds class I MHC”, shown in Figure 19A.
  This reaction shows the first of two steps in the formation of a host‐CoV‐2 complex that has been demonstrated to play a role in immune evasion. In the next step, the output of this reaction is bound by the Beclin‐1 complex and the final complex, “8:class I MHC:BECN1 complex”, is shown negatively regulating the normal human reaction “Capturing cargo and formation of prebudding complex”. This cargo capture reaction is part of the normal “Class I MHC mediated antigen processing and presentation” pathway, as indicated by the flow arrow from the normal reaction to the green‐bordered interactive pathway icon seen in Figure 19A. Users can navigate between the normal and disease diagrams by clicking on the encapsulated pathway icon to open the corresponding pathway diagram. In the normal human pathway diagram, a reciprocal view is shown (Fig. 19B): here, the output of the two‐step human‐CoV‐2 binding reactions is shown negatively regulating the normal reaction in the context of its normal biological pathway and the chimeric complex is associated with the pathway icon for “SARS‐CoV‐2‐host interactions”. Note that pathway connections of this kind are not limited to normal:disease diagrams, but are used throughout Reactome to indicate bridges between any biological processes that are depicted in different pathway diagrams.

**Figure 20:**
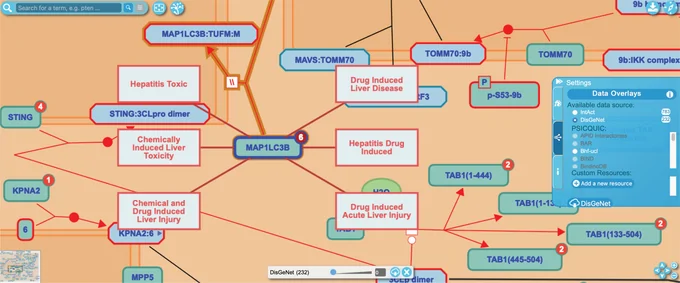
Pathway diagrams can be overlaid with disease associations taken from DisGeNet, as shown here for the protein MAP1LC3B.

11 Reactome supplements its manual disease annotation with an overlay feature that makes use of data from DisGeNet, a public database of associations between human genes or variants and disease (Piñero et al., 2021). This overlay is similar to the protein interactor overlay from IntAct described in Basic Protocol 1, Step 10 and shown in Figure 9.
  To explore this feature within the “SARS‐CoV‐2 host interactions” pathway diagram, open the interactive panel on the right side of the visualization panel, select the middle tab with the data overlay options, and select “DisGeNet” from the available data sources.
  Protein icons in the pathway are now decorated in the upper right with a red circle indicating the number of diseases from DisGeNet that are associated with that protein.
12 Navigate to the reaction “SARS‐CoV‐2 M protein bind MAP1LC3B” under the “SARS‐CoV‐2 modulates autophagy” subpathway and click on the red circle on the human protein input MAP1LC3B to reveal the six associated diseases, as shown in Figure 20.
  Note that the confidence levels may need to be adjusted on the slider bar to reveal all the associated diseases. Clicking on any of the disease icons takes the user to the corresponding record in DisGeNet.

## Basic Protocol 3 FINDING THE PATHWAYS INVOLVING A GENE OR PROTEIN

This protocol will describe how to identify pathways and reactions that involve a gene or protein of interest. For the purposes of illustration, the cyclin‐dependent kinase 7 gene will be used, which has the following identifiers:

**Table:** 

Protein product:	Common name: Cdk7
	UniProtKB (SwissProt): P50613 (CDK7_HUMAN)
Gene:	HGNC: 1778 Entrez Gene: 1022
	GenBank: NM_001799
	Ensembl: ENSG00000134058.

See Alternate Protocol 1 to search by a database accession number rather than by a common name.

### Necessary Resources

#### Hardware

Computer capable of supporting a Web browser and an Internet connection

##### Software

Any modern Web browser such as Firefox, Safari, and Chrome will work to display Reactome Web pages

**Figure 21:**
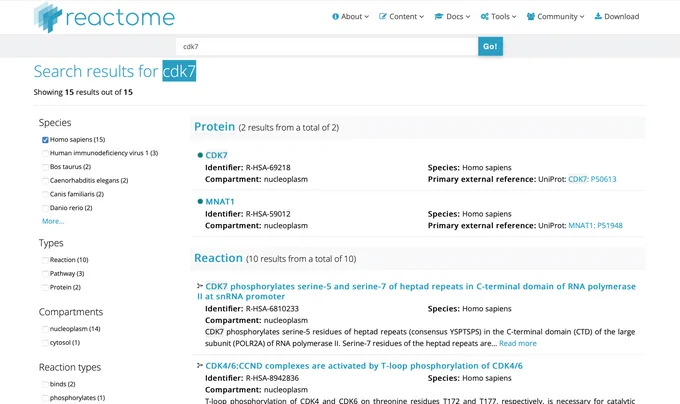
The results page from a simple CDK7 search on the Reactome home page are shown here.

1 Point the browser to the Reactome home page at https://reactome.org.
2 On the home page, in the search bar near the top of the page, click the text box, type CDK7, then press the “Go!” button. After a few seconds, you will be presented with a results page similar to Figure 21.
  Search results are organized based upon record type, i.e., Protein, Reaction, Pathway; users can filter search results according to the criteria listed at the left of the panel (species, types, compartment, reaction types) and also elect to display search results grouped by type (default) or not. The CDK7 search reveals two “Protein”, ten “Reaction”, and three “Pathway” results. Note that when using a text string, the search results find matches for that text in any Reactome data model field. For instance, the second “Protein” hit is MNAT1, which has “CDK7” as part of one of its identified synonyms; similarly, identified reactions or pathways may have the text string “CDK7” in the summation.

**Figure 22:**
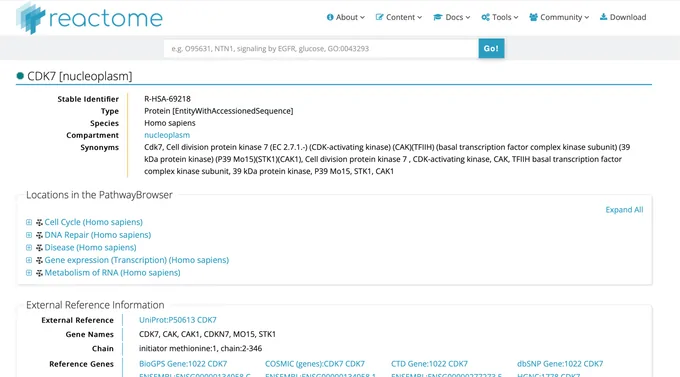
The CDK7 reference entity page.

3 Click on the protein “CDK7” hit from the search results to reveal the page shown in Figure 22.
  This page describes everything that Reactome knows about this protein. In addition to extensive cross‐referencing to external resources, the page identifies all the “Locations in the Pathway Browser” where CDK7 is found in Reactome. Expanding each of the top‐level pathway hits will pinpoint exactly where CDK7 is found in each and clicking on any of the hits will move the user to the relevant pathway diagram. The CDK7 search page also details all the ways that the protein participates in events in Reactome, identifying its role “as an input” or “as a component of”, in this case.
  Clicking on a reaction or pathway hit from the initial CDK7 search reveals a similar summary page for that event, including an interactive reaction or pathway diagram, literature references and editorial attributes in addition to the features shown for an entity described above.

## Alternate Protocol 1 FINDING THE PATHWAYS INVOLVING A GENE OR PROTEIN USING UniProtKB (SwissProt), ENSEMBL, OR ENTREZ GENE IDENTIFIER

Instead of searching for a gene or protein using its common name, as described in Basic Protocol 3, one may wish to use the accession number by which it is known in UniProtKB (SwissProt), GenBank, Ensembl, Entrez, or HGNC. The steps for doing so, using a UniProtKB (SwissProt) accession number, are presented here. The same procedure works for GenBank, Ensembl, Entrez or HGNC identifiers. Note that searching with an identifier rather than a gene name provides more targeted information about the protein of interest but does not identify locations in Reactome where the protein is mentioned in summations or synonyms, as described above in Basic Protocol 3.

### Necessary Resources

#### Hardware

Computer capable of supporting a Web browser and an Internet connection

##### Software

Any modern Web browser such as Firefox, Safari, and Chrome will work to display Reactome Web pages

1 Point the browser to the Reactome home page at https://reactome.org.
2 On the home page, in the search bar near the top of the page, click the text box, type P50613, then press the “Go!” button.
  This brings up the search results page listing the CDK7 protein as the single hit.
3 Clicking on the CDK7 search result loads the reference entity page as shown in Figure 21.
  From here, it is possible to navigate to the pathways and reactions in which Cdk7 takes part, and to view the complexes that contain Cdk7.

## Alternate Protocol 2 USING ADVANCED SEARCH

The simple searches shown in Basic Protocol 3 and Alternate Protocol 1 will suffice for many situations. However, the default search casts a very wide net and may return more hits than one wants. If this is the case, one may wish to use the Advanced Search, which gives much finer control over the search.

### Necessary Resources

#### Hardware

Computer capable of supporting a Web browser and an Internet connection

##### Software

Any modern Web browser such as Firefox, Safari, and Chrome will work to display Reactome Web pages

1 Point the browser to the Reactome home page at https://reactome.org.
2 On the home page under the “Tools” in the Navigation bar, select “Advanced Data Search.”
  The advanced search method permits Boolean‐based queries of the Reactome data set. Combining desired search terms with the appropriate Boolean operators “AND”, “OR”, and “NOT” in combination with quotation marks (for exact term searches), brackets (for grouping terms), the single and multiple wildcard operators “?” and “*”, respectively, and “+” (for “must contain”) or “−” (for “must not contain”) allows users to create precise searches.
  A sample search is preloaded into the search field, querying for Reactome data that contains either the terms “raf” and “map” together, or the term “apoptosis”, or records whose name contains the exact phrase “PTEN S170N” or whose standard IDs are “REACT_1258.1” or “R‐HSA‐198344,1”. This search combination returns 1166 hits when the “SEARCH!” button at the bottom of the page is clicked. Users can also restrict the Boolean search by selecting various criteria from the Filtering Parameters below the search box.

## Basic Protocol 4 USING THE REACTOME PATHWAY ANALYSIS TOOL TO IDENTIFY STATISTICALLY OVERREPRESENTED PATHWAYS

The Pathway Analysis tool allows one to analyze lists of genes, proteins or small molecules by providing services for ID mapping and pathway assignment and overrepresentation analysis. It is a powerful exploratory tool that is linked to the Reactome Pathway Browser. To illustrate how it works, this protocol will describe the analysis of a list of UniProtKB identifiers to identify enriched Reactome pathways.

### Necessary Resources

#### Hardware

Computer capable of supporting a Web browser and an Internet connection

##### Software

Any modern Web browser such as Firefox, Safari, and Chrome will work to display Reactome Web pages

**Figure 23:**
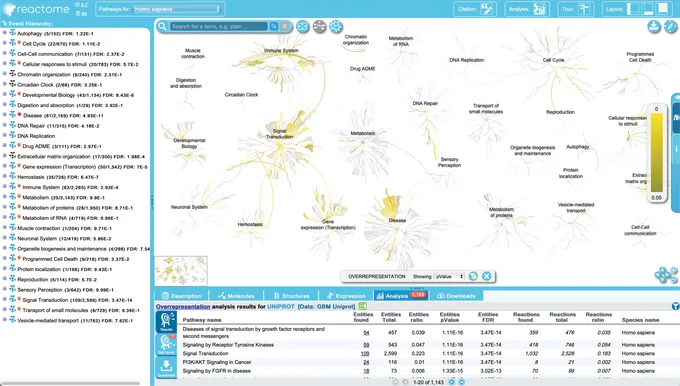
The Pathway Overview showing results of an overrepresentation analysis using the “Analyse Gene list” tool with a list of UniProtKB identifiers.

1 Point the browser to the Reactome home page at https://reactome.org.
2 Click on the “Analysis Tools” button on the home page; alternately, select “Analyse gene list” from under the “Tools” dropdown menu in the home page header.
  This will open a submission form where users can upload sample data sets or user supplied data into any of the Reactome analysis tools listed at the left. “Analyse gene list” is selected by default; other tool options (“Analyse gene expression”, “Species Comparison”, and “Tissue Distribution”) are described in later protocols. Below the tool buttons on the left of the analysis window is a “click to learn more about our analysis tools” option. This takes users to a detailed description of the tools, including some “getting started” tutorials.
3 Select the UniProtKB accession list sample data from the panel at the right of the analysis window and click the “Continue” button.
  The “Analyse Gene list” tool maps a list of identifiers (generally, UniProtKB IDs for proteins, ChEBI IDs for small molecules, and either HGNC gene symbols or Ensembl IDs for DNA/RNA molecules, although other IDs are also supported) to the Reactome pathways that contain them, and overrepresentation and pathway topology analyses are performed. Overrepresentation analysis indicates the probability that the data set contains more of the participants of a given pathway than would be expected by chance, while the topology analysis highlights any reaction whose participants include at least one match to a molecule from the submitted data set.
4 This moves the analysis panel to the “Options” step, where “project to human” is checked by default. In this mode, any non‐human identifiers are converted by the analysis service to their human equivalents. The second option “include interactors” is by default left unchecked; if this option is selected, the analysis will include protein‐protein interactions from the IntAct database for all proteins in all pathways, increasing the potential coverage with the query.
  Leaving these options set at their default values, click “Analyse!” to reveal the overrepresentation analysis overlaid on the Pathway Overview diagram, as shown in Figure 23.
  Note that the color scheme for the analysis may be customized by clicking on the artist's palette on the pop‐out Settings panel at the right. The overview may also be viewed in the space‐filling Voronoi diagram by clicking on the right‐most icon at the top left of the visualization panel.
  A pathway is considered “overrepresented” or “enriched” if the submitted data set has more participants from that pathway than would be expected by chance. The overrepresentation analysis calculates a probability score for each pathway, corrected for false discovery rate by the Benjamini‐Hochberg method (Benjamini & Hochberg, 1995), and colors the pathways according to the scale shown on the right in the visualization panel. Analysis results are shown in the “Analysis” tab in the Details panel. All Reactome pathways are shown, in groups of 20, ranked by the p‐value obtained from the overrepresentation analysis. Visualization may also be set to represent pathway coverage in the query set, by adjusting the toggle in the bottom of the visualization panel.
  The details panel lists the results by pathway for: “Entities found” (number of submitted entities found: clicking on the number in this column will open a new window displaying the identifiers found in that pathway, including mapping to isoform‐specific versions where applicable); “Entities total” (total number of pathway entities); “Entities Ratio” (representing the fraction of entities in the pathway relative to Reactome as a whole); “Entities p‐value” (probability score as described above); “Entities FDR” (p‐value adjusted for multiple comparisons based on the Benjamini‐Hochberg procedure); “Reactions found” (the number of reactions in the pathway containing at least one entity from the data set); “Reactions total” (total number of reactions in the pathway); “Reactions ratio” (fraction of pathway reactions relative to Reactome as a whole); and Species name.
  In the pathway hierarchy panel, pathway names are labeled with the number of molecules from the data set found in that pathway as a fraction of the pathway total, and FDR values are added to the right side of the pathway names. Results from the Topology analysis are also overlaid onto the hierarchy ‐ any reaction in the hierarchy that contains as a participant at least one identifier from the data set is boxed in orange.
  Identifiers from the query set that were not found in a Reactome pathway are listed under the “Not found” tab to the left of the ranked pathways list. This list, as well as the analysis results can be downloaded by selecting the files of choice from the “Downloads” tab to the left of the ranked pathway list. Analysis results are temporarily stored on the Reactome server. The storage period depends on usage of the service but is at least 7 days. Stored results are available via the token assigned to the results file when it is created and displayed in the URL for the results report. The token can be shared and allows later access through the API.

**Figure 24:**
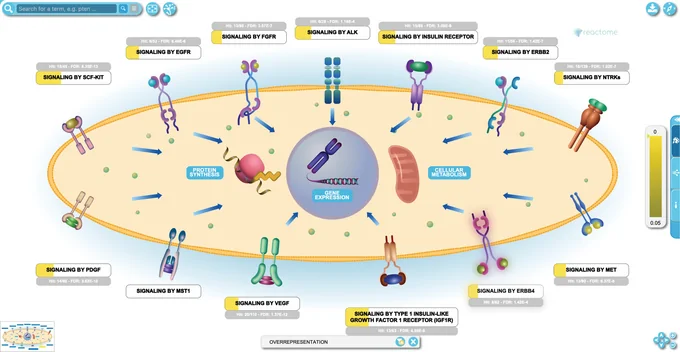
Results of an overrepresentation analysis overlaid on the interactive textbook‐style illustration for the “Signaling by Receptor Tyrosine Kinases” pathway.

5 Results can be filtered to allow users to customize results based on resource (in cases where the data set contains IDs from multiple resources—in this case, this filter is not relevant because all the IDs in the submitted data set are from UniProtKB). Results can also be filtered on the basis of pathway size, species, *p*‐value, and to include or exclude disease pathways.
  Make use of this feature to hide disease pathways from the results, as follows: click on the funnel displayed at the top right of the ranked pathways list in the “Analysis” tab of the Details panel, unchecked the default option “Include disease pathway in the results”, and click apply. The filter can be removed again by clicking on the “x” at the bottom right of the ranked pathway list in the details panel.
6 The analysis view provides an overview of all the Reactome pathways at once. To see the details of a specific pathway, double click on the node representing the pathway in the ranked list or in the hierarchy. To see this, click on the top pathway, “Signaling by Receptor Tyrosine Kinases”.
  This opens the EHLD for the pathway as shown in Figure 24. The yellow band in the subpathway name boxes indicates the proportion of the pathway that is represented in the query data set, while the gray bar above the label indicates the number of pathway entities that are represented in the submitted data, the total number of entities in the pathway and the FDR corrected probability score.

**Figure 25:**
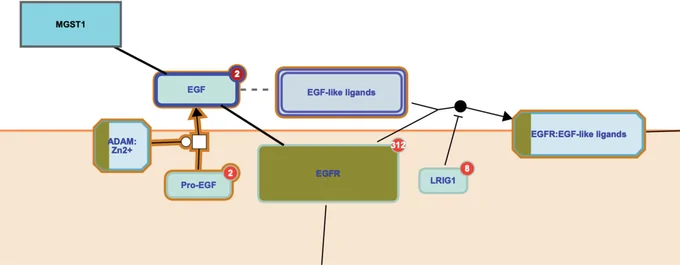
Overrepresentation analysis displayed at the entity level for the reactions in the “Signaling by EGFR” pathway. Interactors for EGF are displayed.

7 Click on the subpathway “Signaling by EGFR” to reveal the reaction‐level diagram.
  In this view, entities that are part of the query data set, such as the protein EGFR, are re‐colored. Similarly, complexes, sets, and subpathway icons are colored to represent the fraction of their components that are represented in the submitted data set. If interactors were included in the analysis, or if the interactor icon on the top right of an entity in the pathway diagram is selected, interactors are also colored to reflect their status in the submitted data, as shown in Figure 25 for the EGF interactors MGST1 and EGFR.

## Basic Protocol 5 USING THE REACTOME PATHWAY ANALYSIS TOOL TO OVERLAY EXPRESSION DATA ONTO REACTOME PATHWAY DIAGRAMS

There are two ways to analyze gene expression data in Reactome. The first method, described in this section, uses an appropriately formatted data set uploaded into the ‘Analyze Gene List’ tool described in Protocol 3 above for Overrepresentation analysis.

The second way to analyze gene expression data in Reactome makes use of the Reactome Gene Set Analysis (Reactome GSA) tool, accessed through the “Analyze Gene Expression” button after “Analysis” is selected from the home page. Reactome GSA performs quantitative pathway analyses, increasing the statistical power of the differential gene expression analysis. This tool is out of scope for this tutorial, but is described in detail in the corresponding publication (Griss et al., 2020) and on the Reactome Web site under “Docs”, “User guide”, “Analysis Tools”, “Analysis Gene Expression”).

### Necessary Resources

#### Hardware

Computer capable of supporting a Web browser and an Internet connection

##### Software

Any modern Web browser such as Firefox, Safari, and Chrome will work to display Reactome Web pages

**Figure 26:**
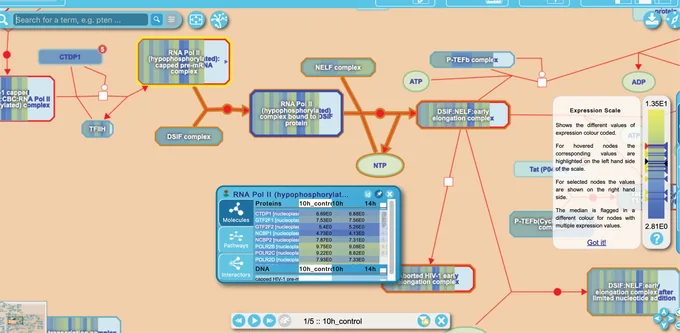
Results from a gene expression analysis using the “Analyse Gene list” tool are overlaid on entities in a reaction from the “HIV Infection” pathway.

1 Point the browser to the Reactome home page at https://reactome.org.
2 Click on the “Analysis Tools” button on the home page; alternately, select “Analyse gene list” from under the “Tools” dropdown menu in the home page header to open the submission form as described in Basic Protocol 3.
3 Ensure the “Analyse gene list” tool is selected (this is the default tool) and click on the sample data set “Microarray data” from the panel at the right.
  Expression data sets are distinguished from overrepresentation data sets by virtue of having multiple columns. The first column must contain the identifiers for the protein, small molecule or other entity, as described in Basic Protocol 3 for Overrepresentation analysis, and the first entry in column 1 must start with the ‘#’ symbol. The remaining columns of the data set must contain only numeric values, with no alphabetical characters. These columns can represent any data that can be inputted as numerical entries: time‐course microarray expression, as in this data set, but also fold change, abundance, or statistical value. Examples include quantitative proteomics, GWAS scores, numbers of somatic or germline mutations (as in the Cancer Gene Census sample data set) or tissue‐specific expression (as in the final sample data set from HPA), among others. Headers for the numerical data columns are supported but not required. For user uploaded data sets, the data should be formatted as a tab‐delimited file, where the first column contains the identifiers and subsequent columns contain the numerical values.
4 Click “Continue” after selecting the Microarray data set, and click “Analyse!” from the Options panel, keeping the default settings of “Project to human” checked and “Include Interactors” unchecked.
  This reveals the results of the analysis, beginning as an overlay on the Pathway Overview display. As above, the user can customize the color selection with the artist palette on the pop‐out Selection panel at the right, and view the results as a Voronoi diagram.
  Pathways are re‐colored according to the numeric values submitted in the test data. The results of the analysis are very similar to those described above for Overexpression analysis, with the following additions:
  ‐ The details panel has additional columns to the right of those described for Overrepresentation, above. These columns contain the submitted expression values or other numerical data.
  ‐ The “Overrepresentation/pathway coverage” toggle in the bottom of the visualization panel has been replaced by a control panel allowing the user to step forward or backward through the columns of data; alternately, the play button may be selected and the series will be shown automatically. The color overlay on the Pathway Overview is adjusted accordingly.
5 Select the top hit from the details panel “Formation of the HIV‐1 Early Elongation Complex” to open the reaction‐level diagram.
  Entities are colored according to their expression (or other numeric) values from the submitted data, and numeric data can be displayed for any affected entity by clicking on the small arrowhead at the right side of the entity icon in the pathway diagram. This opens the contextual information panel (CIP), with molecules, pathways, and interactors tabs; the numeric analysis data is presented in the molecules tab, as shown for a complex in Figure 26. CIPs can be pinned to the diagram by clicking on the pin icon at the top, and multiple CIPs can be opened and pinned at the same time.
  As for “Overrepresentation” analysis results, above, complexes and sets are overlaid with a bar of color that represents the fraction of their components that have expression data in the query set. At a coarse‐grained focus, this appears as a single block of color representing the average expression of all the components. Zooming in on a complex or set reveals individual bars for each component with expression data, as shown in Figure 26. Changes to expression across the time course can be visualized using the forward/backward arrows or the play button at the bottom of the visualization panel; within each complex or set, the bar representing a given component remains in the same position relative to the others. When an entity is selected in the diagram, its icon is outlined in blue as usual (as for the “RNA Pol II (hypophosphorylated complex bound to DSIF protein” complex in the middle right of Fig. 26), and the expression levels of its components are indicated with arrowheads on the right side of the color scale bar at the right of the visualization panel. If an entity in the diagram is hovered over, its icon is shaded in yellow [as for the “RNA Pol II (hypophosphorylated):capped pre‐mRNA complex” at the upper middle in Fig. 26] and the expression values of its components are indicated with arrowheads on the left side of the color scale bar.

## Basic Protocol 6 COMPARING INFERRED MODEL ORGANISM AND HUMAN PATHWAYS USING THE SPECIES COMPARISON TOOL

The comparative analysis of pathways and biological processes offers important information on their evolution and supports metabolic engineering and the study of human disease. Reactome uses manually curated human pathways to electronically infer equivalent events in 15 other species. The Species Comparison tool allows users to compare the predicted model organism pathways with human ones to find pathways conserved (or not) between both species.

### Necessary Resources

#### Hardware

Computer capable of supporting a Web browser and an Internet connection

##### Software

Any modern Web browser such as Firefox, Safari, and Chrome will work to display Reactome Web pages

**Figure 27:**
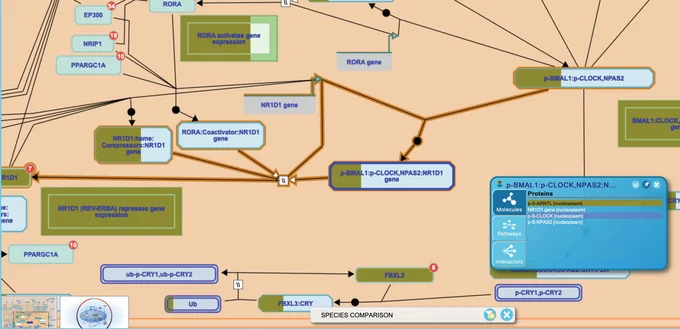
Results from the Species Comparison tool, showing conservation of entities and events from the “Circadian Clock” pathway in mouse.

1 Point the browser to the Reactome home page at https://reactome.org.
2 Click on the “Analysis Tools” button on the home page and click on the third tool “Species Comparison” to launch the data selection page for the Species Comparison Analysis. Alternatively, select “Species Comparison” from under the “Tools” dropdown menu in the home page header and click on the “Species Comparison” button to the left of the analysis window (note that the “Analyse gene list” tool is selected by default).
3 On the “Species Comparison” page is a selection tool that reveals a drop‐down list of species. Select species *“Mus musculus”* from the drop‐down menu and click the “Go!” button to reveal the Reactome‐wide pathway conservation data.
  The “Species Comparison” results are overlaid on the Pathway Overview with pathways colored by the p‐value for entities conserved (as determined by the protocol for computationally inferred events, described here), or by % coverage, depending on which metric is chosen from the dropdown at the bottom of the visualization panel. The color of entities for which no inference was made is left unchanged. Unlike the other tools, the “Species Comparison” analysis infers only on the basis of protein entries with UniProtKB IDs. Small molecules, DNA and other entities that do not have a UniProtKB ID are not considered and are not colored in the analysis.
  The details panel lists the results of the analysis by pathway for: “Entities found” (the number of mouse proteins inferred for that pathway ‐ clicking on the number in this column opens a mapping file that identifies the mouse proteins in that pathway by UniProtKB ID and provides the corresponding human UniProtKB); “Entities total”(the number of human proteins in that pathway); “Entities ratio” (the ratio of proteins from the selected species (here, mouse) in that pathway as a proportion of the total mouse proteins inferred across Reactome as whole); “Entities p‐value” (the probability score for the pathway); “Entities FDR” (p‐value adjusted for multiple comparisons based on the Benjamini‐Hochberg procedure);“Reactions found”(the number of reactions in that pathway containing at least one inferred protein); “Reactions total” (the total number of human reactions in that pathway); “Reactions ratio” (the number of mouse reactions from that pathway as a fraction of the inferred mouse reactions across Reactome as a whole); and “Species name”.
4 Click on “Circadian Clock” in the pathway hierarchy to open the reaction‐level diagram. Entities are colored according to their conservation in mouse as described above. Clicking on the small arrowhead at the right of the icon for a set or complex reveals the Contextual Information Panel (CIP), which provides inference detail on each of the components of the entity, as shown in Figure 27.

## Basic Protocol 7 COMPARING TISSUE‐SPECIFIC EXPRESSION USING THE TISSUE DISTRIBUTION TOOL

Currently, reactions in Reactome represent events that occur within a generic human cell. To facilitate analysis of tissue specific expression, protein expression data has been imported from Human Protein Atlas for overlay on Reactome data. The HPA data reflects the expression of the protein‐coding genes in 44 different human tissues and can be visualized through the “Tissue Distribution” analysis tools.

### Necessary Resources

#### Hardware

Computer capable of supporting a Web browser and an Internet connection

##### Software

Any modern Web browser such as Firefox, Safari, and Chrome will work to display Reactome Web pages

**Figure 28:**
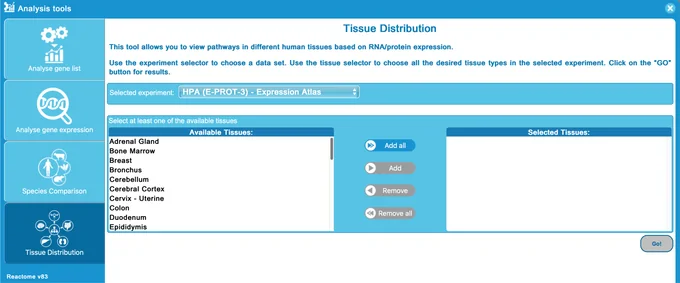
View of the data selection panel in the Tissue Distribution tool.

1 Point the browser to the Reactome home page at https://reactome.org.
2 Click on the “Analysis Tools” button on the home page and click on the fourth tool “Tissue Distribution” to launch the data selection page for the analysis. Alternatively, select “Tissue Distribution” from under the “Tools” dropdown menu in the home page header and click on the “Tissue Distribution” button to the left of the analysis window (note that the “Analyse gene list” tool is selected by default).
  This will reveal the window shown in Figure 28. There is currently one experimental data set available from the dropdown list HPA (E‐PROT‐3). Below the dropdown is an interactive table listing the 44 tissue and cell types from HPA in the left panel. Users can select any of the available tissues and cells by clicking on them in the left panel and then clicking the “add” button from the middle panel. This will duplicate the tissue or cell name in the panel on the right side of the window. Users can also choose to “Add all”, “Remove”, or “Remove all” to tailor the list to their satisfaction. Once the appropriate tissues are selected, the analysis is initiated by pressing the “Go!” button.

**Figure 29:**
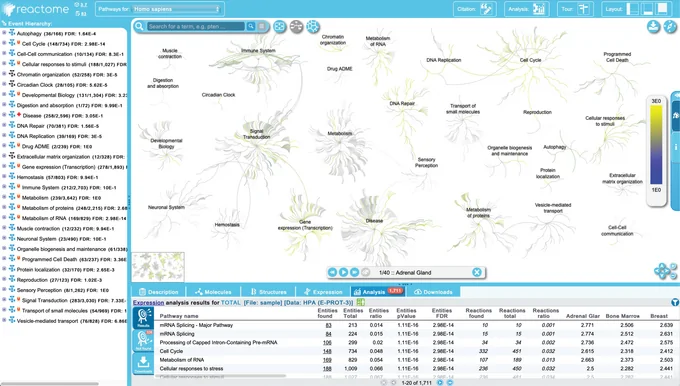
Pathway Overview display of the Tissue Distribution analysis results.

3 For this protocol, select all the tissues using the “Add all” button, and then click “Go!”.
  This will generate the analysis results page as shown in Figure 29, beginning at the Pathway Overview. Pathways are colored according to numeric values submitted in the query data set, as indicated on the scale bar at the right of the visualization panel. The standard details panel is supplemented with additional columns to the right listing the selected tissues and the average value for the submitted identifiers in that pathway for that tissue. Users can view the results for each tissue or cell type by clicking on the forward and backward arrows on the Experiment Browser toolbar at the bottom of the visualization panel.

**Figure 30:**
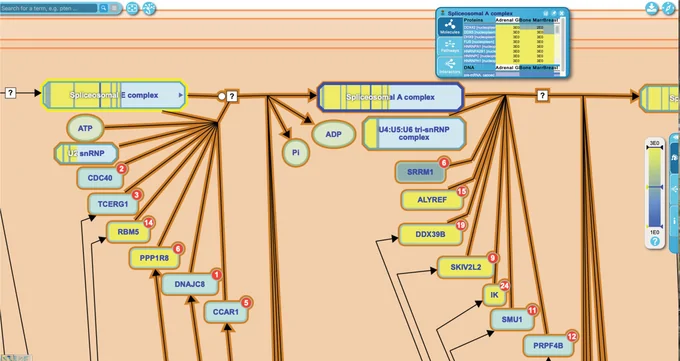
Results of a Tissue Distribution analysis displayed at the entity and reaction level on the pathway “Processing of Capped Intron‐containing Pre‐mRNA”.

4 Click on “mRNA Splicing ‐ Major Pathway” to open the reaction‐level diagram.
  Objects in the Pathway Diagram are re‐colored according to the numeric values from the data set, shown on the scale at the right side of the visualization panel. Entities that are not represented in the input data are not re‐colored. Clicking the forward and backward arrows or the play button in the Experiment Browser toolbar allows users to step through the results for each of the selected tissues and cells.
  As for the “Gene Expression” analysis described in Basic Protocol 4, complexes and sets are overlaid with colored bars corresponding to the proportion of their components represented in the data set, as seen in Figure 30. At a coarse‐grained zoom, the color represents the average of all the components of that set or complex; zooming in reveals individual bars for each constituent represented in the data set.
  Details of the components of a complex or set can be visualized by clicking on the small arrowhead at the right of the entity icon to open the Contextual Information Panel (CIP). Multiple CIPs can be opened and pinned so they remain visible when other entities are selected.

## COMMENTARY

### Background Information

The Reactome project is a collaboration between the Ontario Institute for Cancer Research, NYU Langone Health, the Oregon Health and Science University, and the European Bioinformatics Institute that aims to collect detailed information on all human pathways (Gillespie et al., 2022; Jassal et al., 2020). Reactome pathways are manually curated from the scientific literature by PhD‐level curators and peer‐reviewed by external experts in the field before being released to the Web site. Reactome's comprehensive data model allows for the detailed capture of molecular level events and all annotations are extensively cross‐referenced to other databases and ontologies. All assertions are backed by experimental evidence, either directly from human systems or manually inferred from experiments in model organisms when there is high‐quality protein similarity data to suggest that the same reaction is likely to occur in humans. Reactome is a fully open access and open‐source project. All the software developed for use in Reactome is available for download and redistribution, and the data itself is available in a variety of formats. The Download link on the Reactome Web site provides instructions for obtaining data and software. The robustness, high quality and reliability of the Reactome database is reflected in its status as both an Elixir core resource and a Global Core Biodata Resource.

Reactome uses a simple scheme for describing biological pathways in which all molecular interactions are defined as reactions. A reaction takes a series of inputs and transforms them into a series of outputs, where inputs and outputs can be any type of molecular compound. Pathways consist of a series of reactions in a given area of biology that are linked where possible by shared inputs and outputs in a chain of preceding and following events. For those reactions that are mediated by catalysts, the catalyst enzyme and its activity are noted. Reactions are also annotated using the cellular compartment in which they occur, and the data model is additionally able to support tissue‐ or cell‐type‐specific annotations.

Reactome functions both as a human‐friendly online resource, complete with textbook‐style illustrations, human‐readable summaries and Web‐based tools, and as a powerful computer‐readable graph database with content and tools accessible through APIs (Fabregat et al., 2018; Griss et al., 2020; Sidiropoulos et al., 2017).

As of Version 83 (December 2022), Reactome covers 11,442 unique proteins in 14,471 reactions and 2610 pathways. This represents ∼56% of the human genome, a number conservatively estimated by dividing the number of human UniProtKB entries that take part in Reactome reactions by the total number of human entries in the latest Ensembl human genome build. Reactome's coverage is extended by the highly used Cytoscape app, ReactomeFIViz, which augments manually curated information with high quality functional interactions predicted in the literature (Wu et al., 2014). The Reactome FIViz tool supports a suite of analyses, including pathway enrichment, visualization of drug target interactions, disease discovery, and analysis of single cell RNA sequencing data sets (Blucher et al., 2019; Haw et al., 2020; Wu & Haw, 2017; documentation and user guide here).

Reactome is related to several other pathway databases, but has distinct methodologies and aims, and is distinguished from other pathway databases due to its particular constellation of attributes: robust, detailed data model, human‐pathway focus, manual curation, and expert peer‐review, completely open source and open access, and actively maintained.

HumanCyc, NCI‐PID and Panther Pathways (Mi & Thomas, 2009; Romero et al., 2005; Schaefer et al., 2009) are reaction‐centric pathway databases that are similar to Reactome, although the user interface and underlying database technology are quite different in detail. HumanCyc primarily focuses on intermediate metabolism, whereas Panther Pathways and NCI‐PID emphasize signaling pathways. Active curation of NCI‐PID was stopped in 2012. Panther Pathways allows their pathway data, but not their source code or software, to be used and redistributed freely; use of HumanCyc data or tools requires a subscription and/or a license.

The Kyoto Encyclopedia of Genes and Genomes, or KEGG (Kanehisa et al., 2004), features an extensive set of user‐friendly biological pathway maps that are openly available for personal use; however, a license is required for programmatic access, academic and commercial uses. The BioCarta project (http://www.biocarta.com) represents human biology as a series of colorful high‐resolution diagrams. Unlike Reactome or the other projects mentioned, these diagrams are the end product of the project; there is no underlying database. The focus of BioCarta is to be an education and visualization tool, rather than to support data mining and pattern discovery.

Wikipathways (Pico et al., 2008) is a community‐driven pathway database, built upon the foundations of Wikipedia, which allows community members to freely contribute and edit pathway diagrams. In 2016, an ongoing collaboration between Wikipathways and Reactome was initiated that sees Reactome pathways converted into Wikipathway‐compatible formats, extending the coverage of Wikipathways as well as the potential reach of Reactome pathways (Bohler et al., 2016). Another pathway resource, Pathway Commons, integrates pathway and interaction data from twenty‐two databases, including many of those listed here.

The availability of different pathway resources with varied coverage and aims can pose a challenge to a biologist, who faces the daunting task of visiting each of these sites in an attempt to fill in the holes in one database's coverage with information from the others. The BioPAX project (http://www.biopax.org) has improved this situation by creating a standardized file format for representing biological pathways and reactions. Reactome and many of the other pathway databases have committed to exporting their data in BioPAX format. This has enabled databases to exchange pathways and to co‐curate data, thereby accelerating the rate at which the gaps in pathway knowledge are closed.

Reactome, like other pathway databases, accelerates scientific discovery by assisting in identifying patterns in large‐scale data sets that are difficult to discern from simple inspection.

Reactome's visualization and analysis tools help bioinformaticians, bench scientists and clinicians make potentially unanticipated connections between diverse biological domains, leading to new insights and fruitful areas of investigation. These studies reveal the value of pathway databases such as Reactome in uncovering novel relationships and interactions between genes and contributing to translational research by providing insight on potentially clinically actionable targets in disease.

### Author Contributions

**Karen Rothfels**: data curation, writing: original draft; **Marija Milacic**: data curation, writing: review and editing; **Lisa Matthews**: project administration, supervision, writing: review and editing; **Robin Haw**: project administration, supervision, writing: review and editing; **Cristoffer Sevilla**: visualization; **Marc Gillespie**: data curation; Ralf Stephan: data curation; **Chuqiao Gong**: software; **Eliot Ragueneau**: software; **Bruce May**: data curation; **Veronica Shamovsky**: data curation; **Adam Wright**: software; **Joel Weiser**: software; **Deidre Beavers**: software; **Patrick Conley**: software; **Krishna Tiwari**: data curation; **Bijay Jassal**: data curation; **Johannes Griss**: software, visualization; **Andrea Senff‐Ribeiro**: data curation; **Timothy Brunson**: software; **Robert Petryszak**: software; **Henning Hermjakob**: conceptualization, funding acquisition, project administration, supervision; **Peter D'Eustachio**: conceptualization, funding acquisition, project administration, supervision, writing: review and editing; **Guanming Wu**: conceptualization, funding acquisition, project administration, software, supervision, visualization, writing: review and editing; **Lincoln Stein**: conceptualization, funding acquisition, project administration, supervision.

### Conflict of Interest

None declared.

## Data Availability

The data that support the protocol are openly available at the Reactome Web site (https://reactome.org; https://doi.org/10.3180/19341792) and can be downloaded at https://reactome.org/download‐data.
